# A Mind Map to Address the Next Generation of Artificial Photosynthesis Experiments

**DOI:** 10.1002/smll.202501385

**Published:** 2025-04-03

**Authors:** Christian Mark Pelicano, Sonia Żółtowska, Markus Antonietti

**Affiliations:** ^1^ Department of Colloid Chemistry Max Planck Institute of Colloids and Interfaces MPI Research Campus Golm D‐14424 Potsdam‐Golm Germany

**Keywords:** artificial photosynthesis, solar spectrum utilization, quantum yield, photocatalysis

## Abstract

Artificial photosynthesis (APS) is using light for uphill chemical reactions that converts light energy into chemical energy. It follows the example of natural photosynthesis, but offers a broader choice of materials and components, which can enhance its performance it terms of application conditions, stability, efficiency, and uphill reactions to be carried out. This work presents here first the status of the field, just to focus afterward on the current problems seen at the forefront of the field, as well as discussing some general misunderstandings, which are often repeated in the primary literature. Finally, this perspective article is daring to define some grand challenges, which have to be tackled for the translation of APS into society.

## Graphical abstract

This work explores promising directions in artificial photosynthesis (APS), guided by both scientific innovation and industrial demand. These emerging fields include deep oxidation, flow chemistry for cascade reactions, infrared photochemistry, biotic‐abiotic hybrid systems, AI‐powered APS, and environmental reaction engineering. Finally, insights on the importance of chemistry, environmental analysis and economics to translate APS to practical implementation are provided.

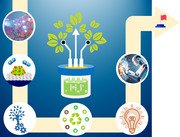

## 1 Introduction: Learning from Nature and the Early Days of Anthropogenic Photosynthesis

Semiconductor photocatalysis has long drawn inspiration by the master‐pattern of natural photosynthesis, developed first in bacteria, then in plants. In nature, light is harnessed in light absorbing and transporting molecules for photoredox reactions, converting otherwise stable products as CO_2_ into intermediates with higher free energy (“uphill reactions”). We can safely assume that most of the surface carbon found on this planet, whether as fossil energy carriers or as more recent peat, humus, or biomass, originated from the uphill photoreduction of CO_2_. Early archaic versions of photocatalysis involved transferring oxidation equivalents from CO_2_ to sulfides (converting them to sulfates) or to iron, where Fe(0) and Fe(II) were oxidized to Fe(III). These processes are kept as an ancient heritage and are still employed as redox mediators in modern biological reaction cascades. When Earth's surface depleted its supply of these electron donors, nature invented as a fix the photosynthetic center II (PSII), allowing from then water to be oxidized to O_2_. We can simplify from that on that natural photosynthesis is able to drive the uphill reaction of converting CO_2_ and water into biomass and oxygen. Human mind is usually attracted by the reasoning and esthetics of nature`s examples, and there is no surprise why the first version of artificial photosynthesis efforts focused on water splitting into H_2_ and O_2_, opting for the simpler H_2_ evolution reaction instead of CO_2_ reduction. O_2_ generation was however not the primary approach, and it is per se difficult to accomplish.

Fujishima and Honda in 1972 demonstrated in their groundbreaking research work that light energy absorbed by semiconductor nanoparticles can split water into H_2_ and O_2_. Their method utilized a TiO_2_ photoelectrode which operates under UV light and required an external bias.^[^ ^1^ ^]^ This discovery sparked widespread interest in photocatalytic water splitting and illuminated the potential of semiconductor materials in solar energy conversion. Since then, there has been remarkable progress in overall water splitting (OWS) via particulate photocatalysis. Over the past four decades, Domen and his group have progressively improved the apparent quantum apparent quantum yield (AQY) of SrTiO_3_ for OWS. In 2020, they reported the development of an almost perfect photoabsorber/cocatalysts system in which nearly every absorbed photon was used in the chemical reaction.^[^ ^2^, ^3^ ^]^ More recently, Wang and his team have reported similar advancements with poly (triazine imide) (PTI/Li^+^Cl^−^), one of the crystalline versions of polymeric carbon nitride,^[^ ^4^, ^5^ ^]^ and reported also AQY near 1.

These two different semiconductor systems are capable of converting UV or blue photons into electron‐hole pairs which are then separated and localized in cocatalysts at distinct positions within the system, achieving nearly 100% efficiency. The localization follows the work function difference between the different photocatalyst's crystal planes.^[^ ^2^ ^]^ Once localized at the cocatalysts, the photoexcited charge carriers can be used for water splitting reactions. These findings prove the feasibility of a near‐perfect artificial photosynthesis. As this “grand challenge” is now accomplished, this article aims to identify the next generation challenges to drive the field toward being applicable for society.

### 1.1 At the Border of Tomorrow: the Current Developments of the Field

#### 1.1.1 Improving Solar Coverage by Slowly Moving Absorption to the Red

**Figure 1:**
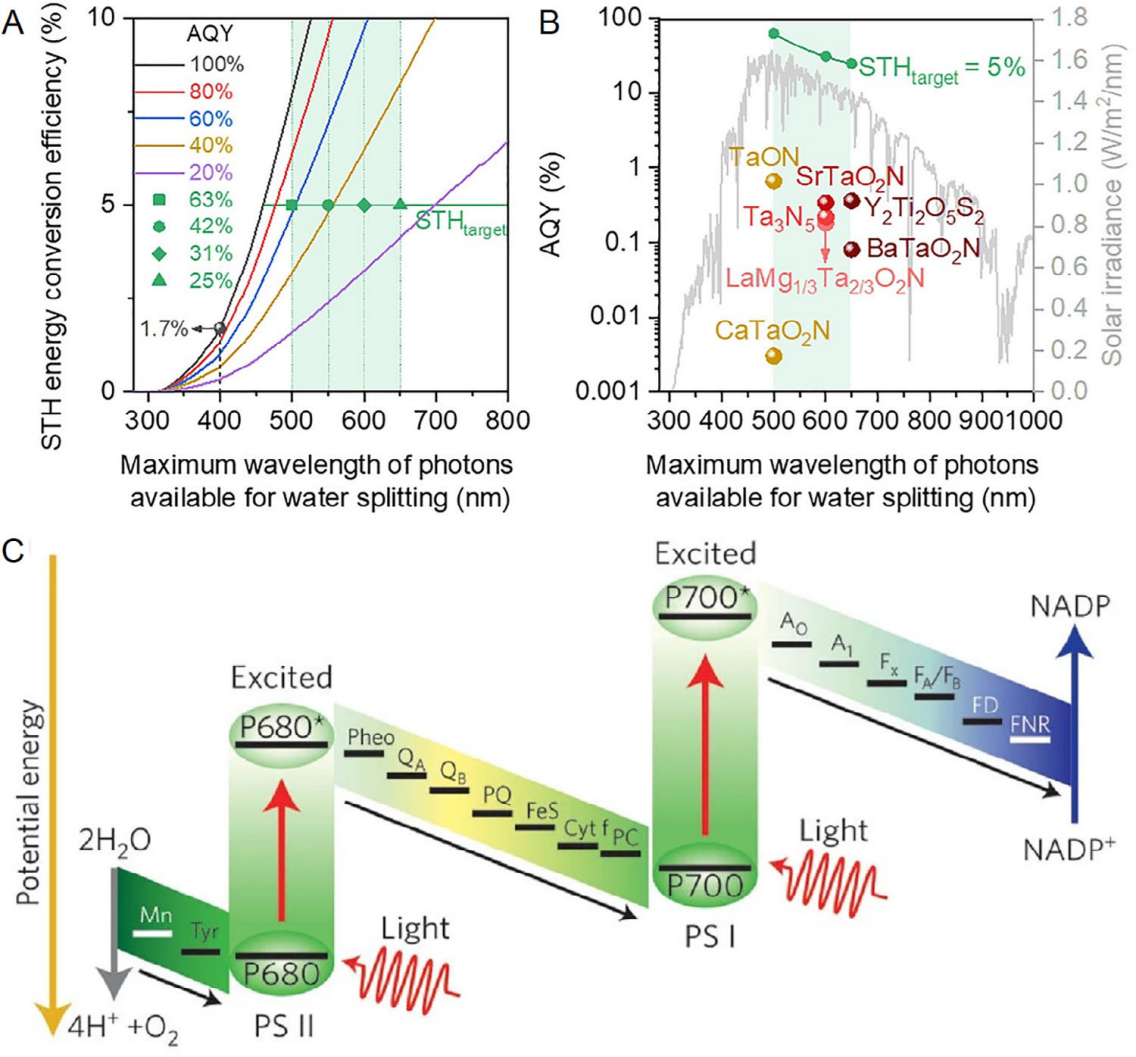
a) Correlation between STH conversion efficiency and photon wavelengths available for photocatalytic one‐step OWS. b) AQY values measured ≈420 nm from one‐step OWS with various oxynitride and oxysulfide photocatalysts, along with the absorption edges of these materials. Reproduced with permission from ref (2). Copyright 2023, American Chemical Society. c) Natural photosynthesis charge‐separation processes, including type I and II reaction centers (simplified Z‐scheme). Reproduced with permission from ref (13). Copyright 2012, Springer Nature.

Techno‐economic studies of OWS indicate a minimum solar‐to‐hydrogen (STH) conversion efficiency of 5%. However, commonly used oxide semiconductor photocatalysts, such TiO_2_ and SrTiO_3,_ which can directly split water, have band gaps larger than 3.0 eV. This restricts their absorption to UV or blue light, restricting their ability to capture a broader portion of the solar spectrum. Hence, it is evident that even with a 100% AQY, this only translates to an STH conversion efficiency of 1.7%, as illustrated in **Figure** **1**a. Since visible light accounts for more than 50% of the solar spectrum, extensive research has focused on developing visible‐light‐responsive photocatalysts. To realize the 5% target, it will be essential to create photoabsorbers with narrower band gaps, ideally with absorption edges at 500 nm or beyond. This to experts’ opinion represents an acceptable compromise between the uses of solar light, while keeping sufficient overpotentials or driving forces for the water‐splitting reactions.

For such materials, maintaining an AQY above 60% would result in a 5% STH energy conversion efficiency.^[^ ^6^ ^]^ As the thermodynamic energy required for water splitting is 4 electrons of E = 1.23 eV, while the overpotential for triplet oxygen evolution reaction can be favorably estimated to be ≈0.27 V per electron, in principle, 4 photons with a wavelength of 826 nm (near IR) should be theoretically sufficient, assuming optimal alignment of the valence and conduction bands. However, photons of higher energy lose excess energy as they relax to the lowest point of the conduction band, wasting that energy. In solar cells, such balances are well elaborated, and the Shockley‐Queisser limit dictates that even the most efficient single‐junction semiconductor cell can reach only 30–40% efficiency.

In inorganic systems, visible‐light‐responsive (oxy)nitrides and (oxy)sulfides semiconductor structures that can absorb visible light up to 600 nm (Figure 1b) hold significant promise as photocatalysts with the potential for high STH efficiencies. For example, a narrow band gap Y_2_Ti_2_O_5_S_2_ photocatalyst (*E_g_* = 1.9 eV), when loaded with Cr_2_O_3_/Rh and IrO_2_ cocatalysts, has been shown to produce a stoichiometric and durable evolution of H_2_ and O_2_ gases, still with a rather low STH efficiency of 0.007%.^[^ ^7^ ^]^ Another example is SrTaO_2_N (*E_g_* = 2.1 eV), a perovskite‐type oxynitride semiconductor with a favorable band structure for promoting OWS under visible light. This material combines the advantages of both oxides and nitrides, offering superior stability in air and moisture when compared to pure nitrides, while featuring smaller bandgaps relative to similar oxides. For instance, by lowering the defect density during SrTaO_2_N synthesis with NaOH and loading RuIrO*_x_* cocatalysts to facilitate charge separation and surface reactions, this OWS system attained an STH efficiency of 0.0063% and an AQY of 0.34%, however at 420 ± 30 nm.^[^ ^8^ ^]^ These AQY values remained low owing to substantial carrier recombination losses at grain boundaries. Further refinement is possibly achievable by adopting the design principles of the SrTiO_3_ photocatalyst as a model. Integrating a molten salt‐assisted synthetic route, along with inclusive precursor selection and aliovalent doping, may unlock new (oxy)nitride or oxysulfide photocatalysts with desirable features. Moreover, producing nanoscale single‐crystal particles with anisotropic facets that possess distinct work functions, as well as selectively placing H_2_ evolution (HEC) and O_2_ (OEC) evolution cocatalysts on specific planes to promote spatial charge separation is a proven way to push the limits of their STH efficiencies.^[^ ^9^, ^10^ ^]^

In carbon nitride photocatalysis or for organic covalent semiconductors, a promising strategy to broaden the absorption spectrum and enhance exciton dissociation is the *incorporation of donor‐acceptor (D‐A) structures* into the system. The motif of D‐A modification is well established in organic photovoltaics and can be transferred –due to the similar physical underlying principles– to APS. For example, a high AQY of 60% (still at 420 nm) for sacrificial H_2_ evolution was obtained by synthesizing triazine‐heptazine‐based carbon nitride by calcining a polymeric carbon nitride in NaCl/KCl eutectic mixture. This system describes an internal donor‐acceptor heterostructure that improves interfacial charge transfer efficiency.^[^ ^11^ ^]^ The same group reported a poly(heptazine imide) (PHI) material produced through the co‐condensation of urea and oxamide, followed by post‐calcination in molten salt.^[^ ^12^ ^]^ This D–A‐copolymerization strategy led to the emergence of new absorption band in the region from 462 nm to 700 nm. Optimized samples in the presence of NaCl exhibited AQY values of 57% and 10% at 420 and 525 nm, respectively, for H_2_ evolution half‐reaction.

Nature is improving spectral coverage by using 8 red photons instead of 4 blue photons in a so‐called Z‐scheme heterojunction. We believe this reflects the evolutionary development of PSII as a late addition (Figure 1C) to promote the move from sulfide to water oxidation,^[^ ^13^ ^]^ necessitated by the hard‐to‐accomplish requirements for oxygen liberation. The solution was to introduce a second photosynthetic system that consumes an additional 4 electrons. *S‐ or Z‐scheme heterojunctions composed of two semiconductors* have been also applied to APS^[^ ^14^ ^]^ and would develop their maximum profitability when the semiconductor bands are complementary, then even potentially surpassing the one‐material Shockley‐Queisser limit. However, this advantage comes at a cost of a factor of 0.5 due to the doubled photon number. To our opinion, while heterojunctions can raise the red sensitivity when needed, they often come with larger trade‐offs in terms of achieving very high efficiencies.

The heterojunction concept is also realized in multijunction solar cells and in photosynthetic bacteria on the colony level, where the semiconductor transducers are arranged in order of color absorption, starting with blue and moving to red. This approach opens the door to more complicated *hybrid designs*, such as an APS layer of blue carbon nitride combined to a silicon solar cell, essentially summing up the efficiencies of both devices.^[^ ^15^ ^]^ As the electric energy might be used for a different purpose (as asymmetrically activating a different chemical state), the “8‐electron problem” might be bypassed.

#### 1.1.2 Moving to More Favorable Products than H_2_ and O_2_

Although simple water splitting is the nearby thought, it is far from being simple. H_2_ is an explosive, low density gas hard to store and transport, while O_2_ not only comes with the triplet overpotential and a minimum loss of about 0.27 eV, but generates also a chemical product otherwise freely available.

Focusing on CO_2_ reduction reaction (CO_2_RR) rather than H_2_ generation is thereby one possibility, as it targets in a biomimetic fashion more stable and long‐lived energy carriers and organic molecules as the products of APS. Challenges related to reaction selectivity, the production of higher‐value products (such as C_2+_ compounds), and overall energy conversion rates are currently addressed for photochemical CO_2_ reduction to emerge as a practical solution for solar energy storage and CO_2_ utilization. Similar to OWS, designing highly efficient catalysts for CO_2_RR via photocatalysis is more challenging than for electrocatalysis or thermocatalysis, as it requires additional attention to factors such as light absorption and charge dynamics. Nevertheless, knowledge gained from electrocatalysis can be transferred to photochemical systems to improve both activity and selectivity. Utilizing metal‐complex electrocatalysts as active sites for photoactive semiconductors has been proven to obtain higher CO_2_ conversion efficiencies and selectivity.^[^ ^16^ ^]^ For example, covalently grafting a Co–quaterpyridine molecular complex to mesoporous graphitic carbon nitride (mpg‐C_3_N_4_) enabled CO_2_ reduction to CO with 98% selectivity and long‐term stability.^[^ ^17^ ^]^ Successful continuous flow hydrogenation of CO_2_ to CO with AQY of 7% at 365 nm was achieved using the CoTiAl mixed oxide at a temperature of 200 °C.^[^ ^18^ ^]^ Photothermal effect of Co‐doped hydroxyapatite catalyst was exploited in continuous flow CO_2_ hydrogenation to CO. Under optimal conditions 62 mmol·g^−1^·h^−1^ of CO at 1 sun illumination and 400 °C was produced with catalysts stability lasting 90 h.^[^ ^19^ ^]^

The possible 2‐electron oxygen reduction gives another high‐value product, H_2_O_2_ (please note that one mole of electrons gives 1 g H_2_, but 17 g of H_2_O_2_, or 23 g of formic acid, and chemistry products are traded by weight). H_2_O_2_ is one of the 10 most produced chemicals on the planet and acts as bleaching agent and disinfectant, with broader applications only restricted by its currently still rather high costs. 2‐electron reduction of O_2_ seems to be an inherent strength of carbon nitrides, and the reported progress is already remarkable.^[^ ^20^ ^]^ Our group specifically focused on ionic carbon nitrides, such as protonated PHI (HPHI) and various metal‐substituted PHIs (M =, Fe^3+^, Ni^2+^, Co^2+^ and Ru^3+^). These materials were prepared via cation exchange with NaPHI and used as photocatalysts for H_2_O_2_ production, with glycerol serving as the sacrificial electron donor. NaPHI and HPHI displayed AQYs of 0.45% and 0.86%, respectively, under 410 nm excitation.^[^ ^21^ ^]^ However, incorporating transition metals into the PHI framework is not favorable for H_2_O_2_ generation; instead, it promotes its decomposition. Coupling KPHI with an adenine‐derived carbon material further increased its AQY to 1.1% at 410 nm.^[^ ^22^ ^]^ More recently, we discovered that incorporating oxamide during the one‐step synthesis of KPHI can induce lattice distortion and activate *n* → π* electronic transition, thereby boosting the photocatalytic efficiency. An optimized sample demonstrated a significant increase in AQY, achieving 41% at 410 nm—substantially higher than the activities of previously reported ionic carbon nitrides for H_2_O_2_ production.^[^ ^23^ ^]^ Another group demonstrated the use of atomically dispersed Sb on carbon nitride for H_2_O_2_ synthesis in a H_2_O and O_2_ mixture under visible light irradiation. This system achieved an AQY value of 17.6% at 420 nm, along with a solar‐to‐chemical conversion efficiency of 0.61%.^[^ ^24^ ^]^

On the photoanode/oxidation side, the pressure for a value preposition is much higher. The standard product dioxygen is not only free of commercial value, but comes only with high energy losses due to the known and well documented oxygen evolution reaction overpotentials. In simple words: although you cannot sell the dioxygen product, you lose a lot of extra energy with it. Any other product which is not spin forbidden in synthesis and valuable could improve this situation. Potential candidates for strongly oxidizing semiconductors are BiVO_4_, WO_3_, α‐Fe_2_O_3_, and carbon nitrides.^[^ ^25^ ^]^

Glycerol or alcohol oxidation in general is only to be considered if the starting alcohol is a side product (such as bioglycerol). Glycerol oxidation in electrochemistry is however rather refined and for instance gives formic acid (as a value product), while providing 8 electron‐proton pairs to the cathode.^[^ ^26^ ^]^ This is meanwhile nicely perfected for electrochemistry, and the therein‐described overpotentials and rates make transfer to carbon nitride photocatalysis very promising. For example, a recent study introduced carbon nitride‐based photoelectrodes that achieved benchmark photocurrent for various oxidation reactions, including those of simple alcohols, ethylene glycol, and glycerol.^[^ ^27^ ^]^

Lignin valorization and polymer upcycling are two promising fields in sustainable chemistry where selective oxidation serves as the primary step to open up the polymer, with the potential to transform waste into valuable resources through innovative photochemical processes. Lignin serves as a rich source of phenolic and aromatic compounds and holds significant potential for high‐value conversion. A groundbreaking advancement in lignin valorization is its transformation into vanillin, one of the most valuable and applied monoaromatic phenols, already produced from lignin at an industrial scale.^[^ ^28^, ^29^ ^]^ The primary method for lignin‐based vanillin production by traditional organic chemistry involves the oxidation of lignosulfonate, with higher yields obtained from the direct oxidation of sulfite black liquor when compared to Kraft lignin.^[^ ^30^, ^31^ ^]^ Kraft pulping on the other hand remains dominant in modern industry, and Kraft lignin—with its lower molecular weight and higher free phenolic hydroxyl content—has been extensively studied for generating high‐value compounds.^[^ ^32^ ^]^ However, due to the limitations of traditional methods, particularly their harsh conditions and the difficulties associated with product separation and purification, there is increasing interest in using solar energy for lignin depolymerization and conversion into valuable products, employing various photocatalysts that function under mild, ambient conditions.^[^ ^33^, ^34^ ^]^ Several systems have been developed to cleave β‐O‐4 linkages in lignin model compounds,^[^ ^35^, ^36^ ^]^ as well as C‐C bonds.^[^ ^37^, ^38^, ^39^ ^]^ These lignin valorization strategies not only provide a sustainable route for producing value‐added products from renewable biomass, but indicate the potential of regrowing starting products with high complexity to set a base for a new chemical industry.

A related topic of similar importance to apply this knowledge to inspire the development of new methods for polymer upcycling.

Polyethylene and polypropylene alone are produced in about 400 Mt/a, with increasing volume, and indeed most of it is not recycled, but ends in waste depots or even in the environment.^[^ ^40^, ^41^ ^]^ “The Great Pacific Garbage Patch,” waste plastic in a closed marine environment, is estimated to contain 150 Mt of waste plastic, alone,^[^ ^42^, ^43^, ^44^ ^]^ which‐ turned back into diesel fuel, would be enough to run 150 million cars for a year. This is only a visible tip of the problem, and for instance polystyrene foam waste or indeed the resins of electronic boards are other hard‐to‐tackle polymer wastes where photochemistry could significantly contribute.

**Figure 2:**
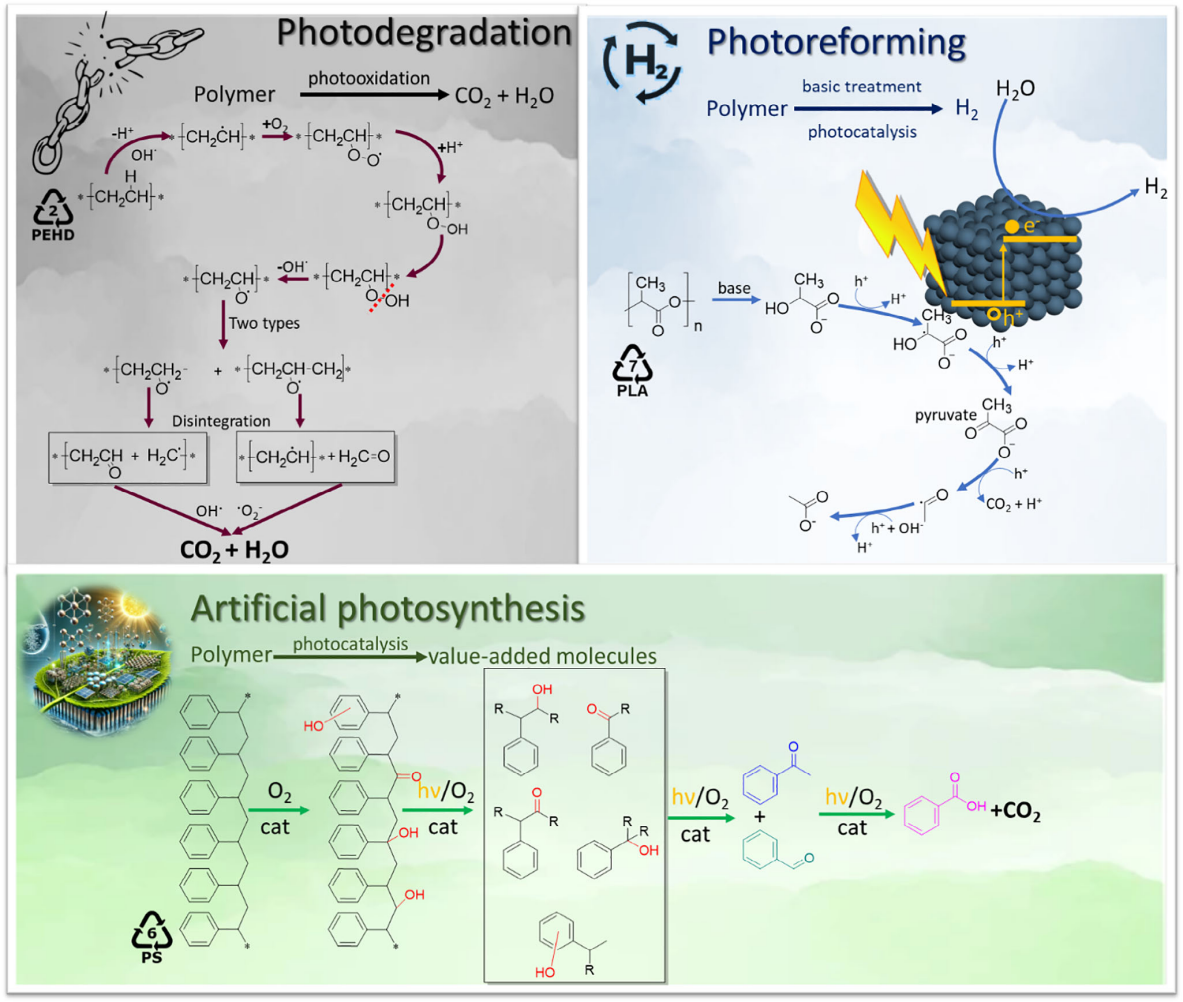
Overview of different pathway for polymer depolymerization using light.

The light‐triggered cleavage of covalent bonds in polymers can be achieved through various methods, based on different fundamental mechanisms and leading to distinct products (see **Figure** **2**). One of the oldest and most extensively studied approach is photodegradation, which involves non‐selective reactions with the goal of calcining polymers into CO₂ and H₂O.^[^ ^45^ ^]^ Photodegradation occurs under radiation of different wavelengths under the help of strong oxidizing agents, like H_2_O_2_ or ozone, and it is essentially a removal strategy for impurities at low concentrations (such as microplastic). Already for this very elemental reaction, achieving reasonable efficiencies within a practical time frame remains a significant challenge, primarily due to the difficult direct activation of polymers and the high binding strength of C‐C linkages.

These classical approaches are however “only” photocatalytic (i.e., they only activate a reaction with already negative reaction potential), while photosynthetic approaches would allow also “uphill reactions,” that is reaction intermediates or the final products could be higher in free energy than the starting polymers, with the necessary energy difference coming from light. This would allow consideration of new reaction pathways or even just accelerated conversion kinetics. Science here moves from only cleaning to “removal and upgrading,” with the idea to turn higher concentration waste streams into valuable products.

Significant advancements in sustainable polymer upcycling were reported by the Reisner group,^[^ ^46^ ^]^ who applied photoreforming to polymers, targeting H_2_ production through the photocatalytic breakdown of polyesters. As this reaction is in most cases energetically uphill, it is indeed a photosynthesis schema rather than only photocatalysis. A complementing approach utilizes materials that absorb solar energy to generate high local temperatures via the photothermal effect.^[^ ^47^ ^]^ The current photothermal reactions only use temperature and are not catalytic, but the increased temperatures could melt the polymers, while a liquid state simplifies mixing and reaction kinetics. Photosynthetic upcycling combined with photothermal processing help is thereby our method of choice, as it could accelerate kinetics while moving products uphill in energy content and value. This concept is however just in infancy and hardly balanced against the other processes.

Only light‐based processes are more commonly described and have recently evolved into a powerful method for directly producing valuable molecules from a wide range of polymer materials, including both hydroxylated (bio)polymers and conventional synthetic plastics.^[^ ^48^, ^49^ ^]^ These products can potentially be integrated into existing chemical processes. For example, Reisner and co‐workers^[^ ^50^ ^]^ developed a mild depolymerization method for polystyrene (PS) using fluorenone as a catalyst and H₂SO₄ as a sacrificial agent under blue LED light. This process successfully broke down PS into small organic molecules, with benzoic acid as the main product (38% yield), alongside other aromatic compounds. In groundbreaking work from Kokotos group,^[^ ^51^ ^]^ PS degradation was performed without acid additives under 390 nm irradiation, achieving a 58% yield of benzoic acid using anthracene as a photocatalyst. These studies paved the way for the direct use of small molecule photocatalysts in selective oxidative polystyrene cleavage with good atom efficiency.^[^ ^52^ ^]^ Moreover, inorganic salts like FeCl₃ form photoactive clusters that can also facilitate PS depolymerization, highlighting the role of both hydrogen atom transfer reagents and radical mechanisms in producing valuable chemical products.^[^ ^53^, ^54^ ^]^ These primary studies rely however on molecular photocatalysis, and their problems such as high operational costs, product separation difficulties, and sustainability concerns, are well described. The future here likely lies in adopting heterogeneous semiconductor systems optimized for water splitting for greener and more sustainable conversion of polymers.

A variety of light‐driven concepts and heterogeneous photocatalytic systems were recently described,^[^ ^55^, ^56^, ^57^, ^58^, ^59^ ^]^ highlighting the largely untapped potential of photochemistry in polymer upcycling. However, several challenges are still to be addressed. For instance, unlike the removal of organic pollutants and heavy metals from water, the insolubility of polymers in water significantly complicates photocatalytic reforming. Additionally, the complexity of the entire process of the mostly applied radical processes should be studied to optimize the use of reaction intermediates or moderators. Designing photocatalysts that selectively cleave specific chemical bonds can enhance the conversion of targeted products and improve the selectivity for high‐value compounds. On the positive side, one has to mention that photosynthetic semiconductor systems are rather inert against dirt, side products, or adherences, and are thereby suited for real waste or also environmental samples. One of the simple reasons for that is that the reactive centers are activated by light, while they are rather unreactive in the dark. Passivation of the catalytic centers by, for example, organic functionality is thereby temporal and can only occur in the light activated state. Lifetime of, for example., carbon nitrides even in contact with full environment of biological species was reported to exceed at least 1000 h.

Integrating photocatalytic technology with other advanced approaches is another way to enhance polymer conversion and have a positive impact on the transformation products of polymer waste. In general, ligninolytic enzymes that are able to cleave C‐C bonds in lignin can break C‐C bonds in polymers. It was proven that manganese peroxidase (MnP, EC 1.11.1.13) and lignin peroxidases (MnP, EC 1.11.1.13) enzymes can degrade polyethylene,^[^ ^60^ ^]^ other microorganisms were documented to degrade PS including *Azotobacter beijerinckii* HM121^[^ ^61^ ^]^ and *Exiguobacterium sp*. strain YT2.^[^ ^62^ ^]^ The combination of enzymatic biocatalysis or utilizing biological funneling methods alongside photoreforming holds significant potential for addressing the challenges of plastic waste.^[^ ^63^ ^]^

If human society is serious on circular economy, the future of polymer waste upcycling lies mandatorily in converting the waste back into (potentially even more valuable) organic molecules. Recent successes in photocatalytic polyacrylate decomposition^[^ ^64^ ^]^ show promise for expanding the range of recyclable plastics, though future efforts are needed to address more and more difficult plastics. Notable example involves resins, where the pioneering work of Knowles and colleagues^[^ ^49^ ^]^ demonstrated the degradation of phenoxy resin and thiol‐epoxy thermoset resins to obtain bisphenol A derivatives. By applying photocatalyzed proton‐coupled electron transfer (PCET) conditions—there still using an iridium photocatalyst ([Ir(dF(CF₃)ppy)₂(5,5′‐d(CF₃)bpy)]PF₆), collidine as a Brønsted base, 3,4‐difluorothiophenol as a hydrogen atom donor, and three equivalents of methanol in dichloromethane under blue LED light. This process yielded the depolymerization product, dimethyl bisphenol A, in 63%. Subsequent O‐dealkylation using tribromoborane successfully regenerated the bisphenol A monomer. Notably, this method enabled the selective depolymerization of hydroxylated polymers while leaving other polymers, such as poly(vinyl chloride), PS, and poly(methyl methacrylate), unaffected, demonstrating its practical applicability. In another work, polystyrene‐based resins,^[^ ^51^ ^]^ including aminomethylpolystyrene resin and Wang resin, were upcycled to benzoic acid with yields of up to 30% using anthraquinone as a photocatalyst under UV light conditions.

Another key area of research is the development of advanced photoreactors, especially flow systems, for large‐scale, effective conversions. In flow systems, the channel can be adjusted to the light penetration depth, making optimal use of the photons, while contact time to the catalysts can be adjusted to the reaction time by the flow rate. That way, flow chemistry accelerates photocatalysis, enabling continuous, scalable, and efficient plastic upcycling while minimizing energy consumption.^[^ ^65^ ^]^ Using high photocatalyst to substrate concentrations, even extreme reaction profiles could be realized, good to avoid consecutive reactions or dynamically bias different reaction pathways. For instance, Savateev et al could realize photo‐flow‐membrane reactor where complete substrate conversion could be realized in contact times below a millisecond.^[^ ^66^, ^67^, ^68^ ^]^ Significant progress in the field was achieved by the Noel group, who developed a robotic platform called RoboChem,^[^ ^69^ ^]^ This platform enables self‐optimization and facilitates the scale‐up of photocatalytic reactions within a continuous‐flow microreactor. RoboChem is designed to handle various aspects of photocatalysis, including hydrogen atom transfer, photocatalysis, photoredox catalysis, and metallaphotocatalysis.

Inspired by the Chinese legend of Yu the Great, who controlled floods by regulating the river's flow instead of blocking it and used the water to create irrigation systems, the scientific community should view polymers and biopolymers as high energy resources. These materials can be transformed from problematic waste into valuable chemicals and fuels using advanced *photosynthetic* systems.

#### 1.1.3 Light: A Diluted Reagent

At this point, we want to discuss a common misunderstanding of photosynthesis, where light acts as a reagent (as at one photon is used for every molecule). The solar constant and the solar productivity are usually comparably low. Using standard photovoltaic cells with an efficiency of about 20%, one can harvest about 360 kWh/year·m^2^, equivalent to 1296 MJ annually. Photocatalysis with the discussed 10% STH thereby generates for instance 4.6 kg dihydrogen, which is for most colleagues unexpectedly low. This hydrogen productivity of course can be recalculated by proton‐electron equivalents in any other organic molecule, not changing the fact that the costs of a photosynthetic device to be economically operational must be very low. In addition, the concept to replace a highly intense chemical factory by a photosynthetic one is simply wrong, as the scale of productivity are orders of magnitude apart. This is why potential application of artificial photosynthesis are also “diluted,” for instance for the decentral production of disinfectants such as H_2_O_2_ on the gram scale for only local use.

Generating larger amounts, we have to compare artificial photosynthesis not with industrial chemistry, but rather with agriculture, where products are generated on the hectare scale. Here, the comparison ends up favorably for artificial photosynthesis: the earlier discussed 10% STH system would generate 46 tons of H_2_/year (or 490 tons of methanol), to be compared with 7.5 tons of, for example, wheat from agriculture.

**Figure 3:**
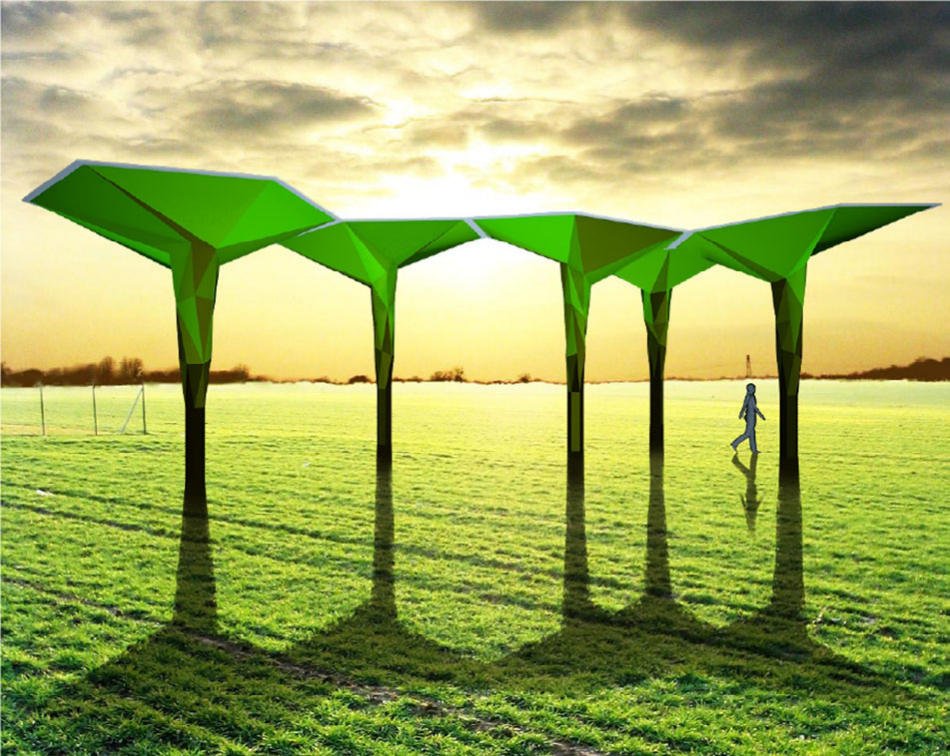
An illustration of an “artificial tree” which can generate value‐added fuels from CO_2_ and water.

Artificial photosynthesis thereby might be an ideal complement to agriculture with a beneficial value preposition, especially for arid regions, as much less water is needed for product generation. **Figure** **3** shows a visionary illustration of such an “artificial tree,” designed to capture CO_2_ and water. Preferentially, the trunk would generate and store high‐value fuels and feedstock, with periodic harvesting throughout the year.

#### 1.1.4 Future: Potential Grand Challenges for Artificial Photosynthesis

“It's tough to make predictions, especially about the future.” This quote of Yogi Berra is of course the more appropriate, the further the future is. Nevertheless, as the seeds of future papers in current publications, it is a tempting operation to try to illustrate some potential future fields of APS, both by “science push” and “market pull.”

### 1.2 Deep Oxidation

Classical chemistry as we know it is restricted on an electrochemical scale by the reductants and oxidants available, plus the stability of reactors and the solvent to apply these conditions. While we can safely state that natural chemistry on planet Earth is occurring around the standard hydrogen potential (RHE = 0), plus that we have for spontaneous reactions to consider the electrochemical window on water as a solvent and the potential presence of oxygen from the atmosphere. This means that we do not expect to find metallic zinc as a natural mineral, nor that we understand H_2_O_2_ as a stable species. These expectations however can be temporally violated under protective layers (such as in zinc or aluminum products) under exclusion of water and most gases as such. Battery electrochemistry uses all these structural and engineering approaches and runs devices between −3.1 Volts (metallic lithium) and + 1.8 Volts (NiO_2_). Classical chemistry is even in closed sub‐compartments still rather restricted to this range of about 5 Volts. Photochemistry now can extend this reaction band, using the temporal nature of the as generated electron and hole pairs and a potential kinetic discrimination between the wanted substrate reaction and unwanted solvent decomposition. For oxidation reactions, we call this “deep oxidations,” and this implemented as inorganic chemistry represents already the final defense line for water factories against dyes or stable antibiotics, to be removed at low concentrations from drinking water. Heterogeneous catalysts can serve as an inspiration for the activation of superoxidants as persulfate (+2.1 oxidation potential),^[^ ^70^ ^]^ and have reached remarkable efficiencies and reaction rates^[^ ^71^ ^]^ for the chemical removal of some resilient water impurities.

For deep oxidation photosynthesis, the active centers to bind and oxidize the substrate are presumably similar, but the energy of oxidation is coming from the photon driven electron‐hole separation, with the oxidation strength of the hole being related to the valence band position of the semiconductor. Typical valence band positions for metal‐PHIs are +2.5 Volts, for ternary BCN semiconductors + 3.2 Volts, and +3.3 Volts for V‐doped WO_3_.^[^ ^72^ ^]^ Reactions with such short lived holes would in principle enable a complete new chemistry, such as the oxidative fluorination with F^−^ ions,^[^ ^73^ ^]^ standard potential + 2.87 Volts, in close analogy with the already existing electrophilic chlorination with seawater or Cl^−^ ions.^[^ ^74^ ^]^ Especially for materials synthesis, this is a very promising pathway toward ultrastable surfaces.

### 1.3 Flow Chemistry for Cascade and Tandem Reactions

Flow chemistry offers a powerful platform to enhance reaction yields, precisely control reaction conditions and pathways, and significantly reduce reaction times. Despite its potential, the use of flow chemistry in multistep tandem and cascade processes remains limited.^[^ ^75^ ^]^ The role of carbon nitride in designing photocatalytic cascade systems is pivotal due to its unique photocharging capabilities and tunable electronic properties. As it was shown by Savateev et al.,^[^ ^76^ ^]^ carbon nitride possesses ability to accumulate charge carriers under illumination directly influences the driving force for hydrogen transfer, here shown for the photocatalytic tetramerization of benzylamine cascade processes. This controlled accumulation allows for the precise modulation of redox potential and ensures stepwise energy transfer across multiple reaction stages. By optimizing the extent of photocharging, carbon nitride facilitates efficient charge separation and minimizes recombination losses, enhancing the overall photocatalytic performance.

In water remediation, a photocatalytic cascade‐flow system was successfully developed to effectively eliminate sulfamethoxazole from model solutions and hospital wastewater.^[^ ^77^ ^]^ An optimized three‐level cascade system, employing a TiO₂‐decorated carbon sponge as the photocatalyst, achieved an 80% degradation efficiency and significantly reduced the chemical oxygen demand, a critical indicator of organic pollutants in wastewater. Flow chemistry, though still underutilized in multistep cascade processes, holds great promise for future advancements in sustainable technologies,

### 1.4 Infrared Photochemistry, Multiphoton Reactions

A field just in the beginning is given by the use of infrared photons for driving photochemical reactions. It is experimentally observed in many cases that reaction with full solar light work more efficient than the same reaction ran with the same flux of defined photons in the blue or UV region, and a variety of explanations, including photothermal effects, is given. Here, we want to focus on possible photonic effects of such phenomena. On top of the usually discussed electronic transitions, the banding of a semiconductor is also includes vibration and rotation transitions, and taking for instance a typical water vibration at 3000 cm^−1^ with 0.385 eV energy already indicates that IR photons in proximity of an excited state charge can massively contribute to reaction activation. Such multiphoton reactions of course depend on the simultaneously presence of an optical and IR photon, which again depends on photon flux and lifetime of the excited states. Remarkably easy cases are photocatalysts where the charge carriers are already trapped in a long living surface state, such as in the previously discussed “dark photosynthesis.” Then, every surface vibration relaxation has the chance to hit an activated or charged state, while shrinking lifetimes of course will create a lower coincidence rate.

**Figure 4:**
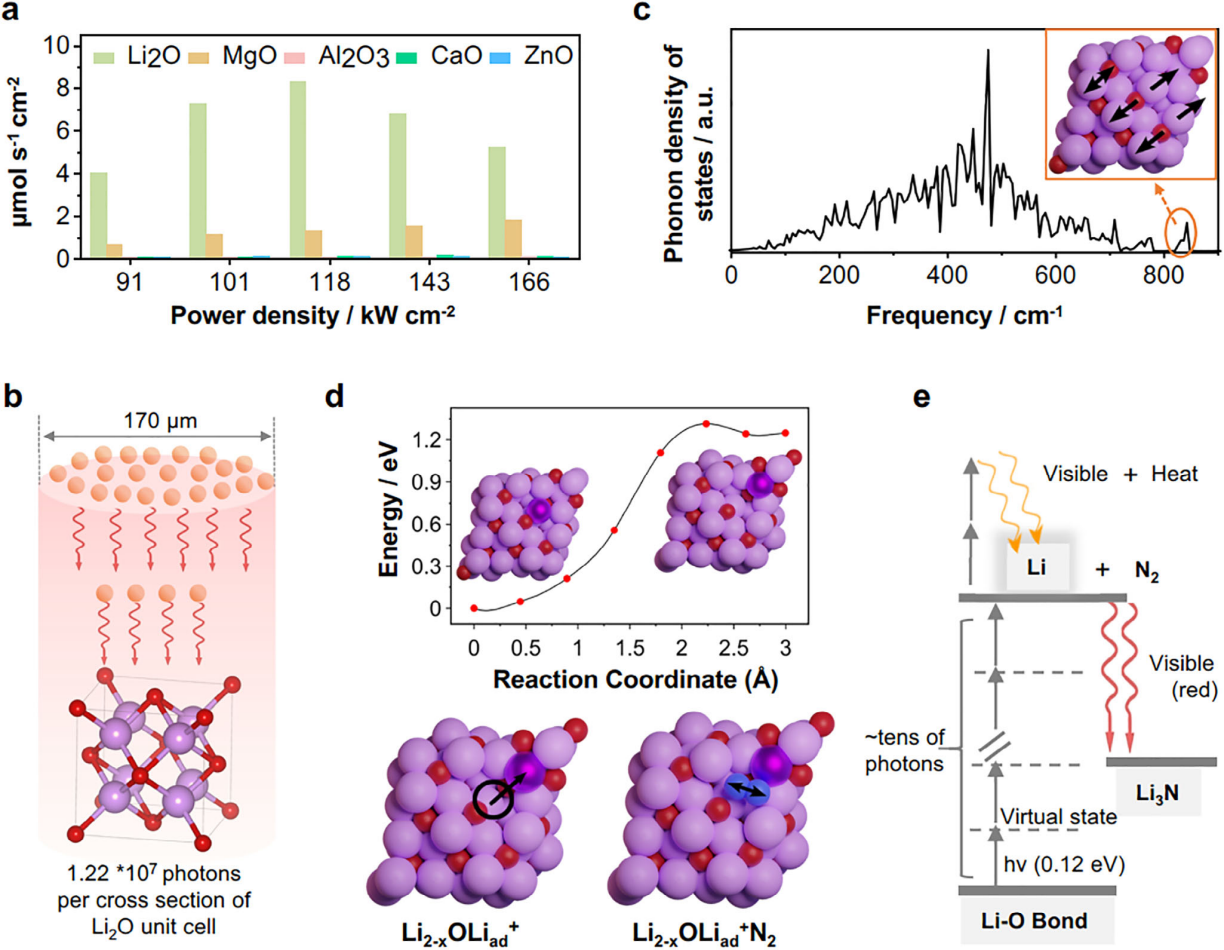
a) Ammonia yield rate by using lithium oxide, magnesium oxide, aluminum oxide, calcium oxide and zinc oxide as precursor mediators: a scanning speed of 0.17 mm s^−1^ and an N_2_ pressure at 7.5 bar. b) The number of photons gathered on a single Li_2_O unit cell (purple ball represents lithium atom; red ball represents oxygen atom) by using laser power of 118 kW cm^−2^ with a pulse (75 µs) and a laser focus diameter of 170 µm. c) The phonon density of states of Li_2_O (211) surface, the inset of the figure shows the atom movements corresponding to high frequency mode (843.7 cm^−1^). d) Nudged elastic band calculation for the generation of the adatom (Li_ad_) defect in the Li_2_O (211) surface. N_2_ adsorption in the vacancy close to the adatom with N‐N bond distance stretching to 1.165 Å (gas phase N‐N  =  1.115 Å). Li adatom is shown in the dark purple color, lithium atoms as purple, oxygen atoms as red, nitrogen atoms as blue. e) Schematic illustration of multiphoton absorption by Li_2_O as an example during the laser‐induced process. After each oxygen‐lithium bond absorbs at least tens of photons (the energy of each photon is 0.12 eV), the oxygen‐lithium bond is dissociated, and the excited state lithium transitions to a lower energy level and emits bright light. Part of the excited state lithium is combined with nitrogen gas forms lithium nitride and emits visible red light Taken with permission from ref. [78].

A model case where multiphoton absorption in the infrared region to drive a photochemical conversion was reported in reference.^[^ ^78^ ^]^ Here, a simple pulsed CO_2_‐laser for engraving was used to excite the stretching vibration of solid Li_2_O with a laser energy of 0.12 eV. This laser energy is not sufficient to drive a chemical reaction, but the combination of high photon flux and sufficiently long vibration lifetimes in the solid state allows multiphoton absorption (of about 30 photons) to make the Li_2_O react with nitrogen from chamber pressure toward Li_3_N. The photochemical nature of the process was then proven with a variety of experiments, including chemical variation, changing the vibration absorption (**Figure** **4**a), as well as a strong red fluorescence obtained from the optical relaxation of the multiphoton saturated excited state throughout conversion (Figure 4b–e). The photochemical overall energy efficiency was calculated to be 3% with respect to the laser energy, an unexpected high value in spite of the very complicated multiphoton process involved. The reported efficiency of the described process is also several orders of magnitude higher than that achieved by simple photothermal heating. This case, however, serves to illustrate that strong photon absorption in the infrared range can indeed contribute to photochemical activity—particularly when it involves eigen‐vibrations of the surface or of adsorbed species.

Apparently, the thermodynamic limitations of traditional one‐photon excitation, such as the restricted redox window, can be resolved by merging the energy of two photons per catalytic turnover. For instance, the introduction of N vacancies in polymeric carbon nitride allowed the degradation of Rhodamine B, methyl orange and water splitting under red‐light irradiation (660 nm).^[^ ^79^ ^]^ Another group elucidated that N vacancies in g‐C_3_N_4_ generate intermediate excitation states, which enables g‐C_3_N_4_ to upconvert long‐wavelength light (800 nm) into short‐wavelength light (436 nm) via a two‐photon absorption process. By pairing this defective C_3_N_4_ with In_2_S_3_ and CdS, an optimal quantum yield of 0.64% was achieved, significantly surpassing the yield of traditional excited absorption modes (≈10^−4^).^[^ ^80^ ^]^ The development of materials for NIR photocatalysis can benefit from the insights and knowledge gained in other fields, such as solar cell technology.

### 1.5 Integration of Microbial Chains into the APS Set‐Up

**Scheme 1:**
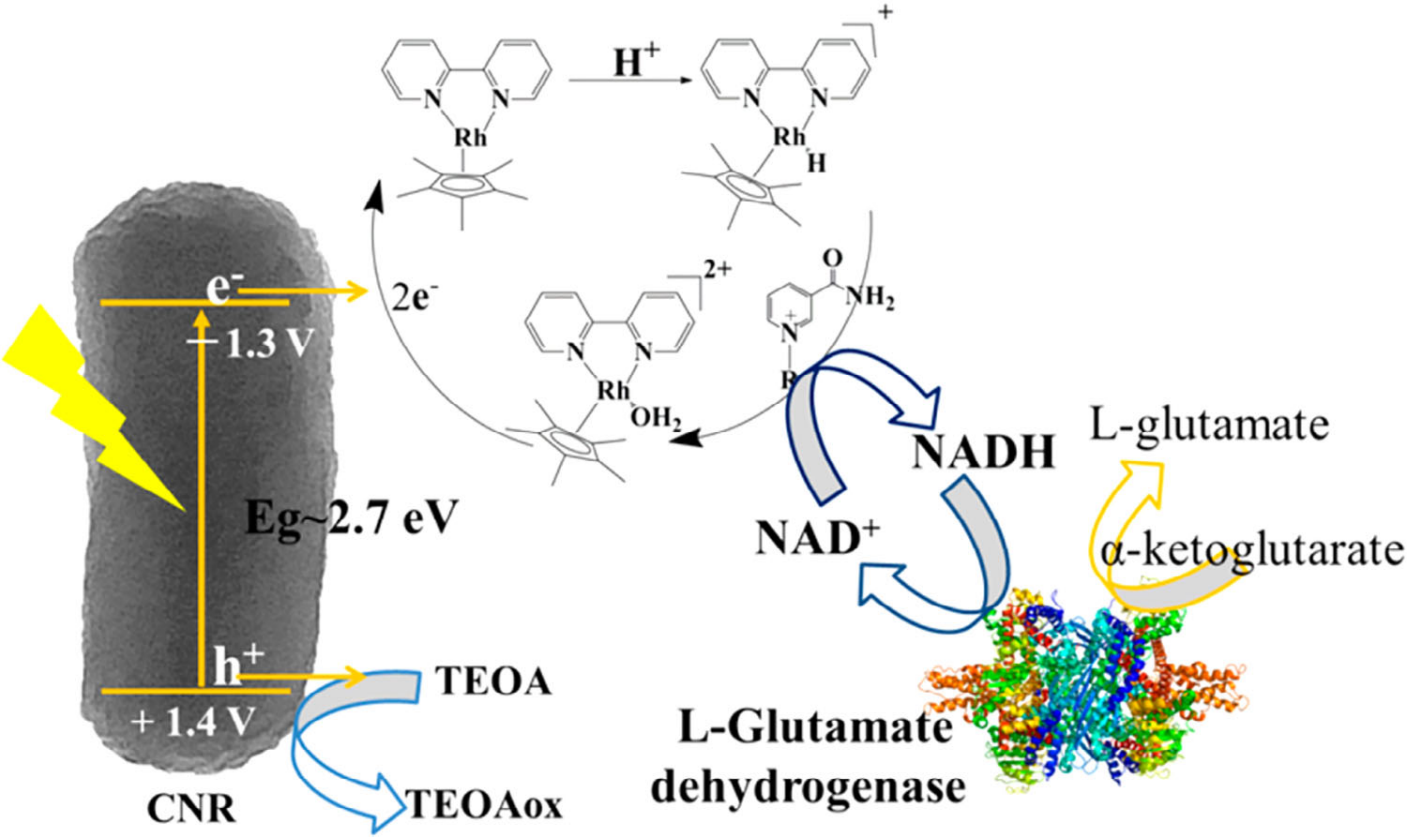
Schematic Illustration of Photocatalytic NADH Regeneration by CNR in the Presence of [Cp*Rh(bpy)(H)]^+^ Acting As Electron Mediator and Hydride Transfer Agent; Regeneration of Catalytically Active [Cp*Rh(bpy)(H)]^+^ is also Illustrated Taken with allowance from ref. [84].

It was already stated that H_2_ is not necessarily an ideal energy storage molecule or transport vector, mostly due to its volatile, low density character. Nature thereby also does not use molecular H_2_, but rather vectorizes H_2_ in reversible molecular carriers, such as the NAD^+^/NADH couple. This reaction is very similar to the standard HER reaction, but even avoids the dihydrogen dimerization (where usually Pt‐group metals are needed) and replaces it by two electron‐proton transfers to the NAD^+^ molecule, or a corresponding further mediator. These simplifications allow NAD^+^ reduction also in APS experiments to be run with significantly improved yields, and reported values exceed 80% AQY. There is a number of publications along these lines available,^[^ ^81^, ^82^ ^]^ and usually, the NADH is immediately used as a substrate of an enzyme to undergo further reaction, such as for the decomposition of peroxides^[^ ^83^ ^]^ or the chiral hydrogenation of α‐ ketoglutarate to L‐glutamate (**Scheme** **1**).^[^ ^84^ ^]^ In the latter case, the enzymatic reaction is so fast that spurious amount of NAD+ could be used, while complete 10 mM conversion of the substrate could be reached in only 8 h. It was calculated that every NAD+ had to undergo 100 redox cycles as a redox mediator, that is, it could be internally recycled.

The costliest factor in such a water – to chiral hydrogen product process is however the enzyme, to be isolated first and re‐added when consumed. To avoid the use of isolated enzymes, hybrid systems that couple photocatalysts with biological machinery presents an innovative approach. The concept of such a combined bio‐approach is to couple artificial photosynthesis of hydrogen carrier molecules with living microbial communities (which are as a system “regenerative” and thereby with potential long‐lifetime). Simplifying APS with electrochemistry, Nocera et al.^[^ ^85^ ^]^ used a solar cell and the generated current for water electrolysis, which as such was metabolized in microbial adlayer to convert CO_2_ into isopropanol and further microbial biomass. The equivalent solar‐to‐biomass yields were reported to be up to 3.2%, well above ordinary photosynthesis of higher plants. The role of the biointerfaces in such “semiartificial” photosynthesis approaches were recently reviewed,^[^ ^86^ ^]^ and we only point to the rich literature referenced in this article. Here, future research could center on the mechanistic understanding of charge dynamics and mass transfer at the interface through comprehensive and multidisciplinary techniques. A transition from inorganic materials to more biocompatible organic semiconductors is also foreseen. Prominent organic photocatalysts include carbon nitrides, COFs, and conjugated polymers can be employed for bio‐photosynthesis (photoconversion).^[^ ^87^ ^]^ An intracellular g‐C_3_N_4_ QDs/NAD^+^ heterojunction has been recently developed, with unique π‐π electron conjugation within living E. coli, achieved a H_2_ generation rate of 7.8 mmol g^−1^ h^−1^.^[^ ^88^ ^]^ As such, bacteria which are otherwise not photosynthetic, are empowered to use light for their metabolism, that is, they become “augmented bacteria.”

### 1.6 Using Artificial Intelligence Approaches in APS

Recent developments in ab initio techniques, such as density functional theory (DFT), have facilitated the rapid design and discovery of new catalysts.^[^ ^89^ ^]^ However, DFT calculations face restrictions in identifying new materials due to limited spatial and temporal resolution, high computational expense, and the sensitivity of exchange‐correlation functionals.^[^ ^90^, ^91^ ^]^ Alternatively, with the vast expansion of available data, artificial intelligence offers a potentially powerful approach to accelerate breakthroughs in key areas, such as APS, including optimizing photocatalyst design, fine‐tuning reaction parameters, and boosting system efficiency for sustainable value‐added chemical production and carbon capture. Machine learning (ML) algorithms can uncover previously unknown relationships or patterns between the materials structures and their catalytic properties,^[^ ^92^ ^]^ facilitating the rational design of high performance and stable photocatalysts at an accelerated pace. For instance, to investigate the structure–property relationship and aid in the development of superior photocatalysts, Zwijnenburg et al. employed a gradient boosting regression model to analyze 6354 conjugated copolymers, successfully identifying new photocatalysts with high H_2_ evolution rates.^[^ ^93^ ^]^ Autonomous precision robotic systems, designed for parallel experimentation within a closed‐loop framework guided by ML algorithms, offer an efficient solution to tackling high dimensionality and sensitivity to synthetic conditions.^[^ ^94^ ^]^ Another promising application of AI is deepening the understanding of critical physical processes in photocatalyst engineering, such as charge carrier dynamics and optimizing visible‐light absorption. In addition, predictive analytics can ensure the long‐term stability and durability of photocatalysts, speeding up the practical deployment of APS systems. Integrating AI into smart reactors with real‐time adaptability to environmental fluctuations can improve solar conversion efficiencies.

### 1.7 Environmental Reaction Engineering

The necessity to remove partly spurious amounts of dyes, pesticides, prions, antibiotics, or other drug molecules was discussed above, and photochemical deep oxidation is certainly a powerful tool. This is potentially also good to attack microplastics or other “inert” polymer water solutes, such as polyacrylic acids or polyglycols. However, whenever a molecule is rather stable against oxidation, such as polytetrafluoroethylene or per‐and polyfluoroalkyl substances, reduction is usually the weak entry to convert. These contaminants where already (falsely) described as “forever chemicals,”^[^ ^75^ ^]^ but also here the art is only to find a path to apply the correct conditions, other than deep oxidation where the C‐F bond is indeed inert against.

For this problem, too, the electrochemical water window excludes electrochemical approaches, but photochemistry with its transient, protected surface states might offer interesting options. For removal by photoreduction, we have to focus on photocatalytic systems with a high conduction band position. For carbon nitrides with their only moderate reduction potential, storage of multiple electrons in one nanocatalyst, followed by PCET is a choice. Here, filling the conduction band with more than one electron (for nanoparticles, easily up to 50 electrons) is generating higher and higher reduction strengths, until the first possible reduction reaction takes place.^[^ ^95^ ^]^ As judged by the possible reactions described, the reduction power of such “loaded” photocatalysts can easily reach the reactivity of NaBH_4_, that is, is quite significant. It is to me mentioned that the loading of photocatalyst with electrons in the light while the reactions in principle can proceed in the dark was also called “dark photosynthesis.”^[^ ^96^ ^]^

## 2 Conclusion

In this opinion article, we gave a personal summary on the current status and perspectives of semiconductor‐based APS, which indeed made remarkable progress in the 50 years after the seminal work of Fujishima and Honda. Nowadays, AQYs of close to 1 have been reported for a diversity of systems and reactions, that is the cascade of light absorption, exciton dissociation, charge transfer of localization to cocatalyst or active sites, and finally highest efficiency for both oxidation and reduction reaction, has been established close to perfection. We can enter thereby a second phase of APS, addressing practical questions and inherent weaknesses of current systems. This includes questions as a more complete coverage of the solar light spectrum to contribute to charge separation, but also the ability to run more valuable reactions than only the splitting of water into hydrogen and oxygen.

A fundamental restriction when using solar light on Earth surface is the diluted character of photons, expressed in moles of electrons per area and time. We are allowed to expect about 4.6 kg H_2_/m^2^year, a dimension too low to compare it with highly intense, centralized chemical processes. This is why we should adopt area needs and cost structure comparable to farming operations, then of course on arid lands or the vast emptiness of oceans. Low density chemical production however goes well with detoxification or the removal of spurious amounts in environmental cleaning.

Important to remember is the primary motivation in advancing artificial photosynthesis – energy generation or synthesis while impacting the environment in a positive fashion, or at least as little as possible. Traditional chemical process design has focused predominantly on access of molecules under maximal profitability. The ongoing environmental crisis necessitates a shift of thinking toward a more holistic approach, incorporating objectives as assessing environmental and societal impacts, or combating global warming.^[^ ^97^ ^]^ Additionally, the rise of microfluidic and flow technologies in environmental science and engineering has become a noteworthy trend, as they allow for precise control of reaction conditions, leading to more effective detection and remediation strategies.^[^ ^98^ ^]^ Thin transparent channel structures also fit ideally to photochemistry, considering light penetration depth, reaction rates, and dynamic control of products and intermediates.

Improving and translating artificial photosynthesis is thereby a multidisciplinary task combining chemistry, reaction engineering, environmental analysis, and economics.

## Conflict of Interest

The authors declare no conflict of interest.

## Data Availability

The data that support the findings of this study are available from the corresponding author upon reasonable request.
